# Supplementary material for: Ultrafast Dynamics of Plasmon-Exciton Interaction of Ag Nanowire- Graphene Hybrids for Surface Catalytic Reactions
Source: Sci Rep. 2016 Sep 7;6:32724. doi: 10.1038/srep32724 (PMC5013321; doi:10.1038/srep32724)
Supplement: Supplementary Information [file srep32724-s1.pdf]

Ultrafast Dynamics of Plasmon-Exciton Interaction of Ag Nanowire-Graphene Hybrids for Surface Catalytic Reactions

Qianqian Ding,[1, 2, 3, +] Ying Shi[4,+] Maodu Chen,[3,+] Hui Li,[4,5,+] Xianzhong Yang,[2] Yingqi Qu,[6] Wenjie Liang,[2] Mengtao Sun[1,2,5,*]

1. Department of Applied Physics, School of Mathematics and Physics, University of Science and Technology Beijing, Beijing 100083, People's Republic of China
2. Beijing National Laboratory for Condensed Matter Physics, Beijing Key Laboratory for Nanomaterials and Nanodevices, Institute of Physics, Chinese Academy of Sciences, Beijing 100190, People's Republic of China
3. Key Laboratory of Materials Modification by Laser, Electron, and Ion Beams (Ministry of Education), School of Physics and Optoelectronic Technology, Dalian University of Technology, Dalian 116024, People's Republic of China
4. Institute of Atomic and Molecular Physics, Jilin University, Changchun 130012, People's Republic of China
5. Department of Physics, Liaoning University, Shenyang 110036, Liaoning, People's Republic of China
6. School of Physics, Peking University, Beijing 100871, People's Republic of China

* Corresponding Author. E-mail: mtsun@iphy.ac.cn (M. T. Sun).

[+] Contributed equally

Table. S1 The fitted lifetimes with the corresponding coefficients of fitting parameters

System	A_1	t_1(fs)	A_2	t_2(ps)
Vis-Ag wire	0.00105	150		
Vis-graphene	-0.00165	275	-0.000439	1.4
NIR-graphene	-0.00254	320	-0.000618	2.5
Vis-Ag wire+graphene	-0.0015	534	-0.0004	3.2
NIR-Ag wire+graphene	-0.00164	780	-0.000454	3.9

The fitted function[26] allows us to fit a kinetic trace for the selected wavelength with a sum of convoluted exponentials:

$$A_1 e^{-\frac{t}{t1}} + A_2 e^{-\frac{t}{t2}}$$

Where A_i and t_i are the amplitudes and decay times (lifetimes), as presented in the table above.

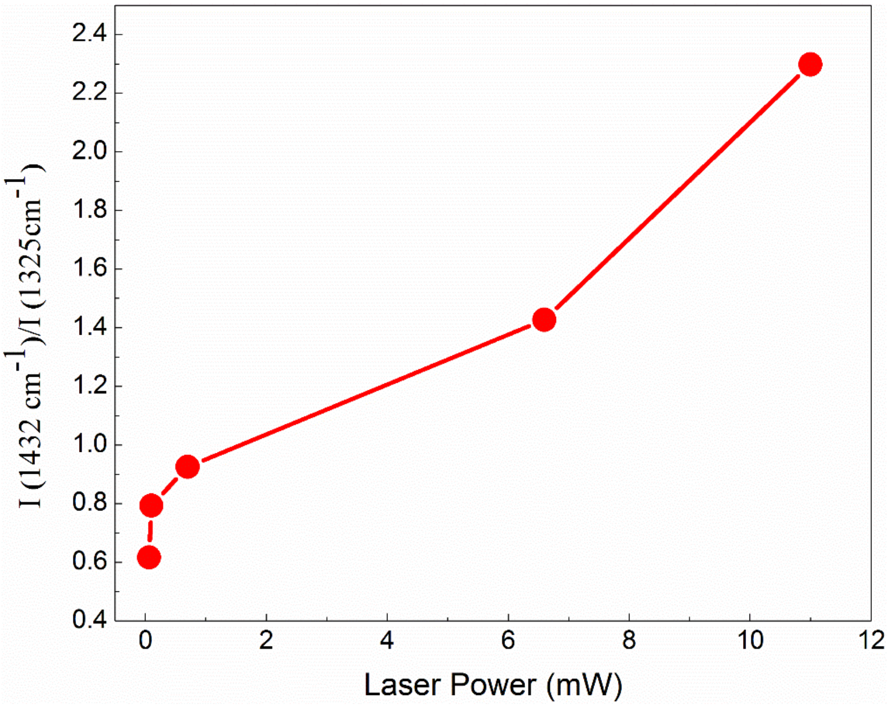

Figure. S1 Relative peak intensity of the band at 1432 cm^{-1} compared to that at 1325 cm^{-1}, plotted against laser power.
